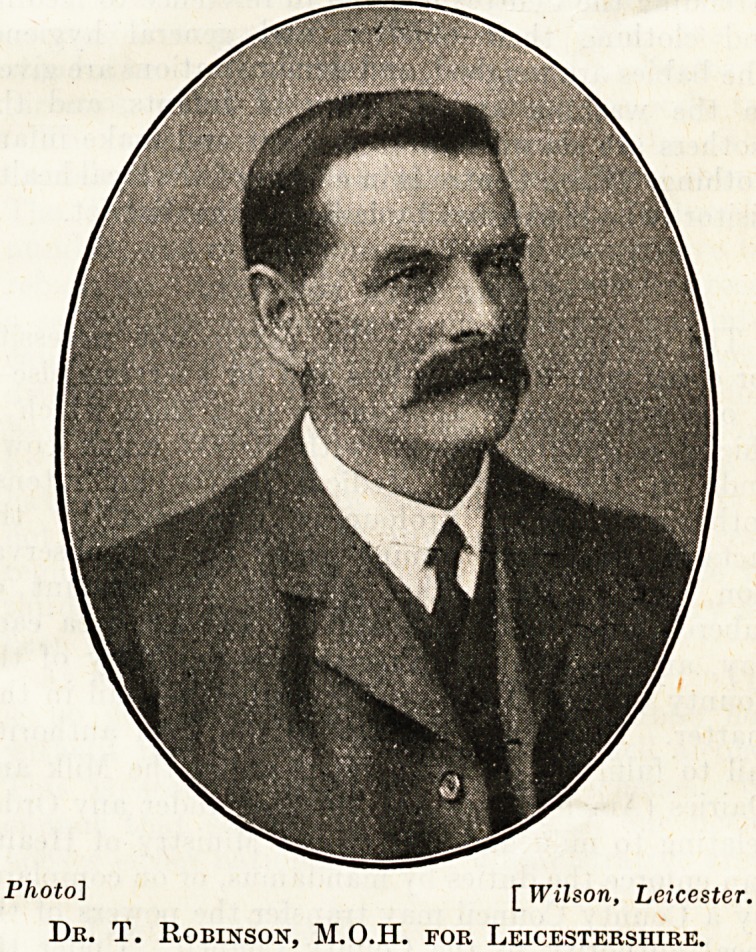# The Public Health: Interviews with Local Authorities—The County of Leicester

**Published:** 1923-10

**Authors:** 


### THE PUBLIC HEALTH.

### INTERVIEWS WITH LOCAL AUTHORITIES.

### XIII.—THE COUNTY OF LEICESTER.

THERE is a well-known picture which, if our memory is not at fault, is called "His Majesty the Baby," in which a policeman holds up traffic, which is both important and considerable, while a baby is taken across the road smoothly and safely. It may serve as an illustration of the precedence which is given to measures for the preservation of child-life by the County of Leicester Sanitary and Housing Committee. The Chairman of that body, Mr. J. W. Black, who extended to us the courtesy of an interview on the subject of the Committee's work, had no hesitation in placing in the forefront of their very varied activities the measures connected with the baby before, at the time of, and after birth. Some five thousand of these important individuals put in an appearance every year within the area of 500,000 acres of which the county consists. The County Medical Officer, Dr. Robinson, whose account of his work was most interesting, also gives pride of place to the fact that the county had the exceptionally low infantile mortality rate of 66 per 1,000 for the year 1922.

*Burton*] [*Leicester.*
ALDERMAN J. W. BLACK, CHAIRMAN OF THE LEICESTERSHIRE SANITARY AND HOUSING COMMITTEE.

*Photo*] [*Wilson, Leicester.*

DR. T. ROBINSON, M.O.H. FOR LEICESTERSHIRE.

#### SAVING THE BABIES' LIVES.

This is indeed a matter for congratulation. This infantile mortality of 66 is 11 per thousand lower than that for Rural England and Wales, which is 77. Moreover, since 1909 the figure for Leicestershire has always been lower than that for the rest of the country. The health visitors were first appointed in 1910, and during the twelve years since then the average infantile mortality rate for this county has been 81, as compared to 92 for the rest of the country. Our attention was drawn to the favourable character of the statistics of particular diseases which relate especially to children—for instance, the death-rate for measles for the county was ·04, as against ·15 for Rural England and Wales; the rate for whooping-cough ·04, as compared with ·16 for the rest of the country; that for infantile diarrhœa 3·26, as compared with 6·2. Special care is devoted to cases of ophthalmia neonatorum, of which there were 26 notifications in the year 1922. As soon as a case is notified the health visitor is communicated with, and she visits and makes a special report on the case. Where a midwife has been neglectful in the course of her duties the case is reported to the Central Midwives' Board. There is strict supervision of midwives attending cases of puerperal fever, and no case occurred in 1922 through the neglect of a midwife.

#### A MIDWIFE FOR EVERY HAMLET.

"In our county," said Mr. Black, "it is our aim to have a midwife available for every birth in every hamlet." Too much importance cannot, he feels, be attached to ante-natal care. As a particular illustration, the report made to the Leicester City and County Councils jointly from the Venereal Diseases Clinics may be quoted. In cases where expectant mothers had been specially treated, in 1922, ten babies were born, and in no case has the child shown any sign of congenital syphilis or gonorrhœal ophthalmia. All these children are being observed at intervals, as well as those born in previous years, and at present no sign of disease has been detected. Work in this direction is bearing out the theory that blindness, deformities and mental deficiency caused by venereal disease can be prevented. The Chairman of the Sanitary and Housing Committee, expressing to us a personal opinion on the production of a clean, healthy race, said that propagation by unfit persons should be stopped by all practicable means, and that he was also personally in favour of a requirement that persons contemplating marriage should produce medical certificates of fitness.

### The Baby Centres.

There was especially brought to our notice the splendid work carried out by the Ladies' Committees of the Infant Welfare Centres, where systematic education by the health visitors of the mothers attending the Centres is given in reference to feeding and clothing their children and general hygiene. The babies are weighed, and demonstrations are given on the washing and dressing of infants, and the mothers are shown how to cut out and make infant clothing. Each Centre is in charge of the local health visitor, who is assisted by ladies in the district.

### Clean Milk for All.

The subject of milk—the paramount necessity for clean milk for the babies and for everyone else—is one which, as is appropriate in a shire which is chiefly a grazing county with 40,000 milch cows, finds in the County Medical Officer an intense enthusiast. He is profoundly impressed by the facts in the county coming under his own observation, and of such other data as the amount of tubercle-laden milk reaching the London area each day, and also by the ultimate responsibility of the County Council if the smaller authorities fail in this matter. He points out that if the local authority fail to fulfil any of their duties under the Milk and Dairies (Amendment) Act, 1922, or under any Order relating to milk and dairies, the Ministry of Health can enforce the duties by mandamus, or on complaint by a County Council may transfer the powers of the local authority to the County Council. Under the 1885 Order and Regulations, local authorities have had the power to enforce the conditions under which milk is produced which are now proved by bacterial examination to be essential for such production—clean udders and teats, hands thoroughly clean and free from all infection and contamination, and milk vessels thoroughly cleansed with steam or boiling water. In the course of lectures to farmers on the milk question, Dr. Robinson has found the more encouraging response from the younger men, and in one notable instance, which he detailed to us, a young farmer went away greatly impressed, set about getting his cows and his cowsheds spotlessly clean, purchased up-to-date appliances, and is now producing wonderfully clean and wholesome milk with the most satisfactory results not only to those who buy the milk, but also—and this is to be specially noted—to his own pocket.

### The Need for Veterinary Inspection.

It would not be right to omit from this review a matter of great importance in which the County Medical Officer has not so far been able to carry his Committee with him. He drew up a scheme for the veterinary inspection of cattle under the Milk and Dairies (Amendment) Act of 1922. Section 5 of the Act prohibits the sale of milk of a cow suffering from tuberculosis of the udder, and a person so offending is guilty if it is proved that he knew, or could by the exercise of ordinary care have ascertained, that the cow was so suffering. The local authority is empowered to appoint veterinary surgeons. Meetings of the district medical officers of health and the veterinary surgeons in the county were held, and it was decided that veterinary inspections of milch cows ought to be carried out four times a year, and that whole time veterinary surgeons ought to be appointed. The County Council is the authority for issuing Grade " A " licences, one of the conditions of which is that a quarterly veterinary inspection of the cows should be carried out. In order to avoid any overlapping of duties and to provide an efficient scheme with the hope of reducing the percentage of samples of milk containing tubercle bacilli and the amount of tubercular disease of bovine origin amongst children, a joint scheme of veterinary inspection was suggested to include all the districts in the county and the County Council—a new voluntary committee of representatives to be formed to work it. It was proposed to divide the expense amongst the urban and rural districts and the county partly on a population basis and partly on the basis of the number of cows in each district. The County Committee, without expressing any opinion, invited representatives from all the urban and rural districts in the county to a meeting to discuss the matter, which was held in June of this year, when nearly all the districts were represented. After a long discussion the following resolution was carried by a large majority :—" That in the opinion of this meeting the duties of sanitary authorities under the Milk and Dairies (Amendment) Act can be more economically carried out by the local authorities than under the scheme suggested by the County Medical Officer." We may perhaps refer to our illustration of " His Majesty the Baby " at the beginning of this article, and venture to say that, in a matter which so profoundly concerns child-life, the set-back to this particular scheme does not imply that Mr. Black and his Committee are not behind Dr. Robinson in his enthusiasm for clean milk.

### Air-space for Bedrooms.

The Medical Officer has some interesting things to say on the matter of air-space in bedrooms in new homes for the workers, to which he thinks that insufficient attention is being given. The County Council had obtained specially from the Registrar-General the returns showing the number of rooms and persons per tenement in each parish in the rural districts in the county. Several copies of these tables were sent to the various rural district councils. They are most instructive and must be an invaluable guide to the local sanitary authorities when considering housing schemes and the size of houses required. The point on which Dr. Robinson rightly lays special emphasis is that the number of cubic feet per adult in the bedroom is of much greater importance from the health point of view than the number of houses per acre, and an extra

£5 spent on increasing a bedroom by 100 cubic feet would be infinitely a greater advantage in the interests of public health than an extra 100 yards of garden. A big air space round the house is of no advantage if the bedrooms are overcrowded and the occupants breathing a vitiated atmosphere continuously for more than one-third of the twenty-four hours. He has recognised for many years that it is imperative that these cases of advanced consumption should be retained in some institution so as to prevent the spread of infection.

### Isolation Hospitals.

The whole of the urban and rural districts of the county have been joined together to form one district for the isolation of infectious diseases under the County Isolation Hospitals Committee. Mr. Black hopes to persuade the Ministry of Health of the need for the erection of buildings at some of the Isolation Hospitals for the isolation and treatment of advanced cased of pulmonary disease, but he is not sanguine as to the ultimate success of such provision until compulsory powers of isolation are obtained.

### The County Laboratory

Space must be found for a brief reference to the excellent work of the county laboratory which was officially opened in 1920. Since that date the examinations have totalled over 2,500 each year. The laboratory is open on Sundays as well as weekdays, and all swabs received on Saturdays and Sundays are examined and the results in cases of urgency are communicated, as on other days, by telephone. During last year 1,093 swabs were examined for traces of diphtheria in the laboratory, which has since its inception examined 2,370 swabs. This shows that the medical practitioners realise the value of a central laboratory, and the diminution in the number of cases of diphtheria and the fall in the mortality during the last three years also indicate its usefulness.

### General Conditions and Death-rate.

Leicestershire, as we have observed, is chiefly a grazing county. Hinckley, Loughborough, Earl Shilton and Barwell are the chief centres for the manufacture of hosiery and boots and shoes. Collieries in the north-west and iron mines in the north-east parts of the county are largely worked, and brickmaking is also undertaken. In the general care of the health of the 250,000 people who occupy this area, Mr. Black and his committee have a task of sufficient variety and magnitude to call for their unremitting care. In addition to the matters on which we have touched, there is the general overriding responsibility of the county in sanitary matters which necessitates constant pegging away at the local districts—the rural districts particularly—to put their sanitation in order. It is uphill work. But that the committee and their medical officer, in their zeal for a proud health record, are getting good results is evident from what we have already seen in regard to the babies, and also from the fact that the general death-rate for the county for 1922 was 11·1 per 1,000, a figure which is 1·8 less than that for rural England and Wales—12·9.

## Figures and Tables

**Figure f1:**
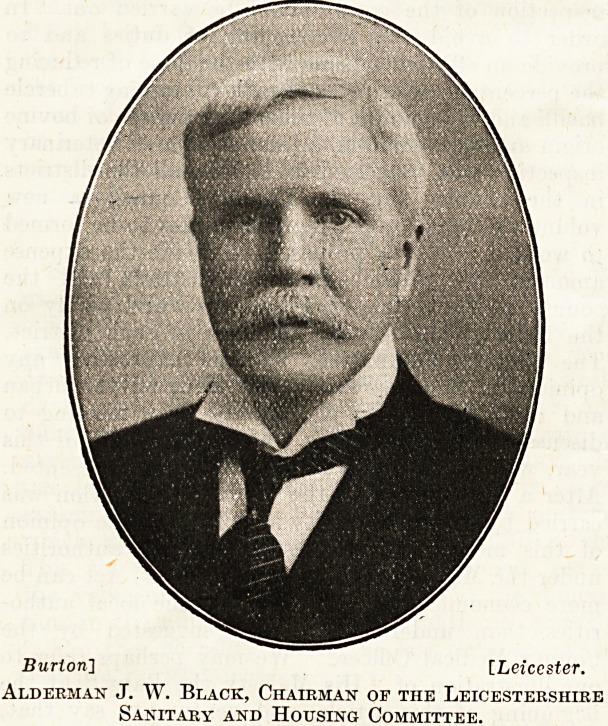


**Figure f2:**